# The artificial neural network approach based on uniform design to optimize the fed-batch fermentation condition: application to the production of iturin A

**DOI:** 10.1186/1475-2859-13-54

**Published:** 2014-04-13

**Authors:** Wenjing Peng, Juan Zhong, Jie Yang, Yanli Ren, Tan Xu, Song Xiao, Jinyan Zhou, Hong Tan

**Affiliations:** 1 Key Laboratory of Environmental and Applied Microbiology, Chengdu Institute of Biology, Chinese Academy of Sciences, Chengdu 610041, PR China; 2 University of Chinese Academy of Sciences, Beijing 100049, PR China

**Keywords:** Artificial neural network, Uniform design, Genetic algorithm, Iturin A, Fed-batch fermentation

## Abstract

**Background:**

Iturin A is a potential lipopeptide antibiotic produced by *Bacillus subtilis*. Optimization of iturin A yield by adding various concentrations of asparagine (Asn), glutamic acid (Glu) and proline (Pro) during the fed-batch fermentation process was studied using an artificial neural network-genetic algorithm (ANN-GA) and uniform design (UD). Here, ANN-GA based on the UD data was used for the first time to analyze the fed-batch fermentation process. The ANN-GA and UD methodologies were compared based on their fitting ability, prediction and generalization capacity and sensitivity analysis.

**Results:**

The ANN model based on the UD data performed well on minimal statistical designed experimental number and the optimum iturin A yield was 13364.5 ± 271.3 U/mL compared with a yield of 9929.0 ± 280.9 U/mL for the control (batch fermentation without adding the amino acids). The root-mean-square-error for the ANN model with the training set and test set was 4.84 and 273.58 respectively, which was more than two times better than that for the UD model (32.21 and 483.12). The correlation coefficient for the ANN model with training and test sets was 100% and 92.62%, respectively (compared with 99.86% and 78.58% for UD). The error% for ANN with the training and test sets was 0.093 and 2.19 respectively (compared with 0.26 and 4.15 for UD). The sensitivity analysis of both methods showed the comparable results. The predictive error of the optimal iturin A yield for ANN-GA and UD was 0.8% and 2.17%, respectively.

**Conclusions:**

The satisfactory fitting and predicting accuracy of ANN indicated that ANN worked well with the UD data. Through ANN-GA, the iturin A yield was significantly increased by 34.6%. The fitness, prediction, and generalization capacities of the ANN model were better than those of the UD model. Further, although UD could get the insight information between variables directly, ANN was also demonstrated to be efficient in the sensitivity analysis. The results of these comparisons indicated that ANN could be a better alternative way for fermentation optimization with limited number of experiments.

## Background

Iturin A is a nonribosomal lipopeptide antifungal antibiotic produced by *Bacillus subtilis*. The iturin A structure consists of two major parts: a peptide ring composed of seven amino acid residues (L-Asn-D-Tyr-D-Asn-L-Gln-L-Pro-D-Asn-L-Ser-) and an 11–12 carbons hydrophobic tail [1-3]. Iturin A is a potential bioresource with broad-spectrum antifungal activity that has been used to treat human and animal mycoses [4]. More recently studies have shown that iturin A could be used as a potential biocontrol agent against harmful plant pathogen that cause crop diseases [5], such as southern corn leaf blight [6] and for controlling fungi in grains [7].

The production of iturin A has gained considerable interest mainly because of its multifarious advantages and diverse potential applications; however, its production on a commercial level has not been successful so far. To increase the yield of iturin A, optimization of the fermentation conditions is one of the primary approaches that can be applied. Fermentation process optimization has been studied intensively by researchers for decades [8], [9]. Traditional optimization approaches are the statistical methods such as the Orthogonal Experiment Method and Response Surface Methodology (RSM) which are used widely on the lab-scale [10]. However, most of these methods require data from a large number of experiments and are not suitable for use on the industrial scale where experimental numbers need to be kept as lower as possible. Therefore, a uniform design (UD) method was developed to meet the industrial needs [11]. Compared with other statistical methods, UD reduces the number of experiments in a multiple-dimension optimization and allows the largest possible number of levels for each factor [12]. UD is an important statistical method that has been used successfully in many process optimizations [13-15].

In the last decade, as a kind of artificial intelligence, artificial neural network (ANN) has been applied to the modeling of non-linear systems, simulating the chaos bioprocess and predicting the results [16,17]. Because ANNs have higher modeling accuracy and generalization capacity, they have become an attractive tool [18]. Additionally, ANN can model all non-linear multivariate functions including the quadratic functions while the statistical methods can only be used for modeling the quadratic functions [19].

ANNs normally require a large number of patterns (experiments) to establish an accurate model, but when the patterns are relatively representative and statistically well distributed, which is the assumed characteristic of statistical methods like UD, ANNs can also establish accurate models with smaller amounts of data. Recent reports indicated that based on the RSM data, the ANN model was more accurate than RSM [17,19-21]. But few studies have reported building ANN models based on UD data, where the experimental numbers were much less than the numbers in RSM. In this work, for the first time an ANN model was established based on UD data and then a comparison between the ANN and UD models was performed to assess their efficiency.

A genetic algorithm (GA) was used to optimize iturin A yield with an ANN model. The GA is an efficient stochastic global optimizing method built on the principles of biological evolution implying survival of the fittest and random changes that has proved to be an outstanding method for multivariable optimizations in biochemical processes [22].

The addition of three amino acids, asparagine (Asn), glutamic acid (Glu), and proline (Pro), to the culture medium during fermentation has been shown to increase significantly the yield of iturin A [23]. In this paper, ANN-GA and UD were used to optimize the yield of iturin A with the same experimental design from UD. Meanwhile, validation experiments using unseen data were carried out to estimate the capacity of both the ANN and UD models as well as the sensitivity analyzing. To the best of our knowledge, this is the first report on optimization by an ANN based on UD data and a comparison of the two methods in antibiotic fermentation.

## Results and discussion

### Experimental design

A three-factor ten-level experimental design with 10 experiments was performed according to the UD table *U*_10_^*^(10^8^). The experimental design and results as well as the predicted outputs calculated by the UD and ANN models are shown in Table 1.

**Table 1:** UD matrix of variables and their experimental responses and predicted values of iturin A titer

**Trail**	**Factor (mg/L)**			**Iturin A titer (U/mL)**		
	**Asn(X**_**1**_**)**	**Glu(X**_**2**_**)**	**Pro(X**_**3**_**)**	**Experimental**	**ANN prediction**	**UD prediction**
1	50	280	140	11866.6 ± 287.8	11866	11928.7
2	65	380	80	10967.8 ± 277.3	10966	10978.9
3	80	260	185	12627.2 ± 279.3	12622	12608.0
4	95	360	125	12429.8 ± 289.6	12419	12458.5
5	110	240	65	12496.7 ± 287.3	12492	12493.4
6	125	340	170	13057.1 ± 282.6	13048	13110.6
7	140	220	110	12604.6 ± 276.3	12602	12625.8
8	155	320	50	12519.1 ± 285.5	12513	12589.4
9	170	200	155	11890.0 ± 274.6	11893	11930.7
10	185	300	95	12706.5 ± 276.4	12702	12694.6

### UD modeling, analysis and optimization

To determine the relationship between the concentrations of the three added amino acids (variables), regression analysis was performed using the Minitab software. A second order polynomial equation, as in Eq. (1), was obtained to correlate the variables with iturin A titer. The results were analyzed by ANOVA, shown in Table 2. The UD model F-value was 105.55, calculated as the ratio of mean square regression and mean square residual, and the model P-value (Prob > F) was 0.009, which is very low. These two values (F and P) implied that this model was significant. The crossed terms, *X*_*1*_*X*_*2*_ and *X*_*2*_*X*_*3*_, were deleted for the high correlation with variable *X*.

**Table 2:** Analysis of variance (ANOVA) for the quadratic uniform design model

**Factor**	**Coefficients**	**Sum of squares**	**Standard error**	**Degree of freedom**	**T**^**a**^	**P**^**b**^
Constant	−4864		1002		−4.86	0.040*
*X*_*1*_	59.97	642299	4.91	1	12.21	0.007*
*X*_*2*_	87.57	6239	8.63	1	10.15	0.010*
*X*_*3*_	21.81	298991	5.45	1	4.00	0.057
*X*_*1*_^2^	−0.23	656908	0.014	1	−15.97	0.004*
*X*_*2*_^2^	−0.15	1445754	0.015	1	−10.10	0.010*
*X*_*3*_^2^	−0.075	111229	0.015	1	−4.95	0.039*
*X*_*1*_*X*_*3*_	0.0011	7	0.028	1	0.04	0.972
Model	F = 105.55 P = 0.009*					
	R^2^ = 99.7 % S = 65.4117					

^a^T denotes t-value.

^b^P denotes P-value.

*denotes significant.

The P-values were also used in the analysis of the independent variables to check the significance of the coefficients, which are necessary to elucidate the pattern of the mutual interactions between them. The P-value of each term, as well as the estimated coefficient and t ratio are given in Table 2. The coefficient was deemed significant when P was less than 0.05, and the smaller the P was, the more significant it indicated. The P-value indicated that *X*_*1*_ (Asn), *X*_*2*_ (Glu), *X*_*1*_^2^ (Asn × Asn), *X*_*2*_^2^ (Glu × Glu) and *X*_*3*_^2^ (Pro × Pro) were the significant terms. Among the independent variables, addition of Asn to the culture medium (P = 0.007) had the most significant effect on the iturin A titer followed by Glu (P = 0.01) and Pro (P = 0.057). All the quadratic terms having small P-values indicated that the quadratic terms also had significant effects on the iturin A titer, and the effects of the variables on the responses were not a simple linear relationship. The interactions between the variables were not significant, and only the interaction between Asn and Pro had a small effect.

The regression equation was as follows:

$$\begin{array}{l} Y=-4864+60.0X_{1}+87.6X_{2}+21.8X_{3}-0{{.226X}_{1}}^{2}\\ \;-0{{.150X}_{2}}^{2}‒0{{.0752X}_{3}}^{2}+0.0011X_{1}\times X_{3} \end{array}$$

where *Y* is the titer of iturin A; and *X*_*1*_, *X*_*2*_ and *X*_*3*_ are the concentrations of added Asn, Glu and Pro respectively.

The fit of model was also indicated by the coefficient of determination R^2^, which was calculated to be 99.7%, suggesting that the model was efficient and could explain 99.7% of the experimental data.

The optimization was based on taking the partial derivative with respect to *X*, which were calculated as 133.1 mg/L, 292 mg/L and 145.9 mg/L for the concentrations of added Asn, Glu and Pro, respectively. This gave the maximum predicted result for the iturin A titer (*Y*) as 13509.1 U/mL.

Three repeat verification experiments were carried out and the average experimental iturin A titer at the optimal feed condition was 13221.7 ± 275.7 U/mL, which was close to the predicted result of 13509.1 U/mL. The prediction deviation was 2.17%.

### Artificial neural network and genetic algorithm

#### ANN modeling and analysis

The same experimental design that was used in the UD method was used as the training set for the ANN modeling, as shown in Table 1. Other experimental data that were not part of the training set were used as the test set (unseen data) to test the generalization capacity of the ANN model, as shown in Table 3. A feed-forward back-propagation (BP) net was chosen with three neurons in the input layer and one neuron in the output layer. The Levenberg-Marquardt Back Propagation training function was chosen and the structure including one hidden layer with seven neurons was decided, which had the minimum mean-square-error (MSE) (0.083 for the scaled data) between the predicted outputs and experimental responses of the test set. Meanwhile, the MSE for the training set was 7.74 × 10^−4^ (for the scaled data). Thus, the selected ANN model had a 3-7-1 topology, i.e. an input layer with three neurons, a hidden layer with seven neurons, and an output layer with one neuron.

**Table 3:** ANN and UD prediction for unseen test data

**Trail**	**Factor (mg/L)**			**Iturin A titer (U/mL)**		
	**Asn(*X***_***1***_**)**	**Glu(*X***_***2***_**)**	**Pro(*X***_***3***_**)**	**Experimental**	**ANN prediction**	**UD prediction**
1	50	200	125	11668.6 ± 276.1	12085	10647.9
2	50	300	50	10763.5 ± 279.8	11481	11255.8
3	50	380	180	10850.6 ± 277.3	10439	10696.4
4	125	200	50	12100.5 ± 277.0	12128	11533.6
5	125	300	125	13064.1 ± 275.9	13074	13451.9
6	125	380	180	12570.8 ± 274.9	12381	12245.0
7	180	200	180	11856.0 ± 171.4	11778	11656.8
8	180	300	125	12878.3 ± 272.9	12950	12968.4
9	180	380	50	12363.2 ± 268.6	11863	11153.5
10	100	333	200	12215.8 ± 272.7	12068	12787.5
11	175	233	175	12143.9 ± 274.1	12288	12527.9
12	50	400	150	10108.0 ± 274.4	9910	10197.3
13	200	200	100	11922.0 ± 275.6	11675	11066.0
14	75	366	75	10914.9 ± 282.2	11232	11551.1
15	150	266	50	12244.3 ± 271.2	12532	12649.5

When the ANN model was built, its fitting capacity and generalization capacity were measured by analyzing the experimental data and model prediction data of the training set and test set, respectively. The average error for the training set and test set were 0.039% and 2.19%, respectively. The correlation coefficient between the model-predicted results and experimental results was 99.998% for the training set and 92.618% for the test set. The small MSE and average error and the high correlation coefficient for the training set indicated that the ANN model processed outstanding approximation ability. Further, the satisfied values of MSE, average error and correlation coefficient for the test set suggested that the ANN model also had good generalization capacity.

#### Sensitivity analysis

**Figure 1:**
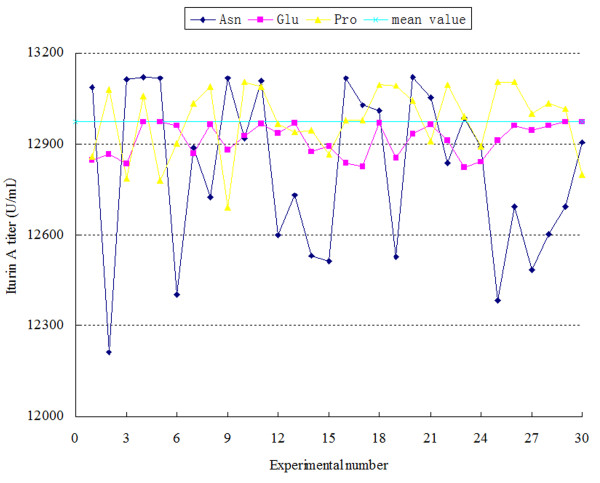
**Sensitivity curves of inputs to outputs based on mean value.** It indicated the effects of each independent variable when changing around their mean values.

A sensitivity analysis for the ANN model was performed to estimate the effects of input variables on the predicted iturin A titer. The analysis was carried out on the ANN model decided above. Because the ANN model cannot directly give the relationship between input and output, a perturbation analysis based on the mean value of the input data was used to elucidate such insights of the system. Thirty random perturbations of each variable were tested. The pertuibations fluctuated around the mean value of the input data by the amplitude of the standard error. The sensitivity estimation value was calculated as *S* in Eq. (6). The perturbation curve for each variable and the corresponding *S* value are shown in Figure 1 and Table 4. The *S* values for the concentrations of added Asn, Glu, and Pro were 271.1, 54.8 and 114.4, respectively. The sensitivity curve for variations in the concentrations of added Asn had the biggest fluctuation range around their mean values, compared with the fluctuations in the sensitivity curves for variations in the concentrations of added Pro and Glu. This result indicated that the concentration of added Asn had the most significant effect on the titer of iturin A, followed by the concentration of added Pro and Glu, when the variables perturbed around their mean values.

**Table 4:** Sensitivity analysis of variables to outputs

**Variables**	**Amplitude of perturbation**	***S *** **value**
Asn addition concentration	45.4	271.1
Glu addition concentration	60.6	54.8
Pro addition concentration	45.4	114.4

**Figure 2:**
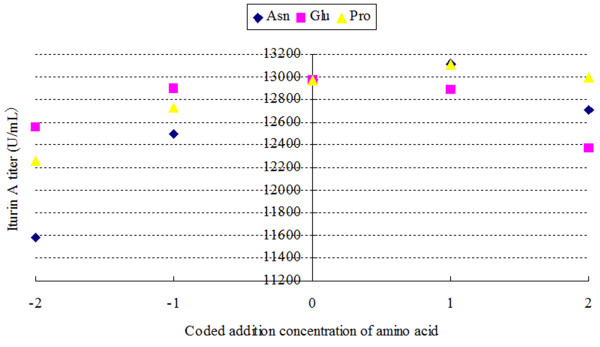
**Sensitivity analysis of ANN model using perturb method.** It indicated the effects of each independent variable when changing in the entire optimized range. The coded values were shown in Table 5.

Interestingly, when an ‘perturb method’ [19] was applied over the entire range of input data using coded values, the sensitivity changed close to the edge. As shown in Figure 2 and Table 5, each series in the graph represented the changing rate of outputs caused by the variation of variables; the higher the slope, the greater the effect of the variable. When the perturbation emerged near the mean value (coded as 0, range from −1 to 1), the sensitivity of each variables was comparable to the results shown in Figure 1. However, when the perturbation emerged near the edge (range from −2 to −1 and 1 to 2), the slopes of curves of the Asn, Glu, and Pro were 660.5, 428.5 and 292.0, respectively, which indicated that the concentration of added Asn had the most significant effect, followed by Glu and Pro, on the iturin A titer. This result suggested that the model could not be explained simply by a linear relationship because the sensitivities were different within different input ranges.

**Table 5:** coded values of variables

**Variables**	**Coded values**				
	**−2**	**−1**	**0**	**1**	**2**
Asn	50	83.75	117.5	151.25	185
Glu	200	245	290	335	380
Pro	50	83.75	117.5	151.25	185

#### Optimization based on GA

A GA-based method was used to optimize the input variables with the goal of maximizing the iturin A titer. The ANN model was used for the fitness function of GA and the optimization process was run several times with different random initial populations. These repetitions ensured that the global optimum to which GA converged under most of the initial conditions was obtained. Based on this ANN-GA optimization process, the maximum iturin A titer was predicted to be 13257 U/mL when the concentrations of added Asn, Glu, and Pro were 155 mg/L, 320 mg/L and 160 mg/L, respectively. Three repeat verification experiments were carried out under the feed condition described above, and the experimental result for the iturin A titer was 13364.5 ± 271.3 U/mL, which was reasonably close to the ANN-GA prediction. Based on this result, the predictive error of the ANN-GA method was 0.8%. The iturin A titer increased significantly by 34.6% from the control (9929.0 ± 280.9 U/mL) in which the batch fermentation was performed without the addition of the three amino acids.

#### Comparison of UD and ANN-GA

The same experimental design was used to train the UD and ANN models and the comparison was based on the correlation coefficient and the root-mean-square-error (RMSE). The fitting values (prediction) of ANN and UD model are shown in Table 1. Both models had excellent fitting accuracy. The ANN model had a smaller deviation (RMSE) than UD model (Table 6), suggesting that the ANN model had the better approximation capacity.

**Table 6:** Comparison of predictive capacity of UD and ANN

	**Training data**		**Validation data**	
	**UD**	**ANN**	**UD**	**ANN**
RMSE^a^	32.21	4,84	483.12	237.58
C^b^	99.86%	1.0	78.58%	92.62%
error %	0.26	0.039	4.15	2.19

^a^RMSE is the root-mean-square-error.

^b^C is the correlation coefficient.

**Figure 3:**
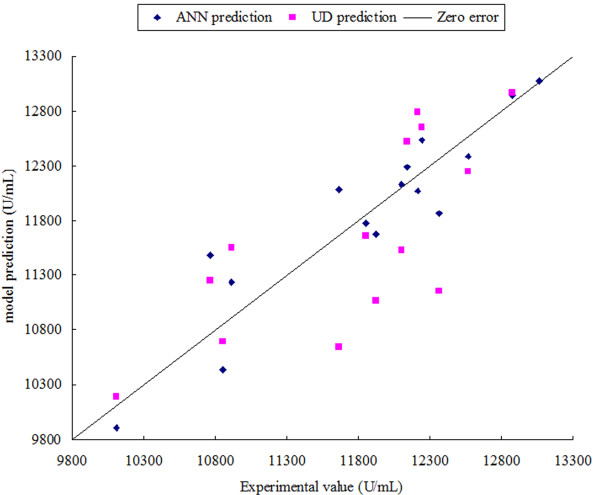
**Comparison of generalization capacity of UD and ANN model.** It showed the parity plot for ANN and UD prediction for the unseen data.

Generalization capacity is an important parameter that is used to evaluate predictive models. Generalization capacity can be estimated only by using input data that is different from the training data set, therefore, 15 additional experiments were carried out to generate a test data set (shown in Table 3). The UD and ANN models were both tested using the new unseen test data. The experimental and predictive results are listed in Table 3. The RMSE between the experimental response and the result predicted by the UD and ANN models were 483.12 and 237.58, respectively, and the correlation coefficients for UD and ANN were 78.58% and 92.62%, respectively. Figure 3 shows the comparative parity plot for UD and ANN predictions. The deviations for both UD and ANN can be observed directly from the figure. The ANN model fitted the experimental data more accurately than the UD model prediction, which showed larger deviation than ANN. The RMSE for ANN was about two times less than the RMSE for UD and the correlation coefficient was higher for the unseen data by ANN, indicating that the ANN model had significantly better generalization capacity than the UD model. The higher predictive ability of the ANN model is likely to be because of its universal capacity to approximate any non-linear situation, while the UD model is limited to the second order polynomial.

In the sensitivity analysis of the UD model, the significance of the variable effects on the iturin A titer can be observed directly from the equation and the ANOVA analysis, and based on these, the addition of Asn was found to be the most significant factor followed by addition of Glu and Pro. By contrast, for the ANN model, its sensitivity of the input variables cannot be obtained directly, but several methods are available to obtain insights into the relationships between inputs and outputs. In the perturbation analysis based on the mean value of the input data, when the perturbations occurred around the mean values, Asn addition was the most significant factor followed by Pro and Glu addition. In the perturb method based on the entire range of the input data, close to the edge of the range, the significance was Asn, follow by Glu and Pro, which interestingly was quite comparable with the sensitivity analysis results for the UD model. Thus, ANN was also efficient in the sensitivity analysis.

The optimal results for UD and ANN-GA were compared. Although under optimal conditions their experimental values were not statistically significantly different, the prediction error of ANN-GA was only 0.8%, which was quite a bit less than the 2.17% error of UD. Therefore, the optimization ability of ANN-GA was demonstrated to be better than UD.

These comparisons indicated that both the ANN and UD model had satisfactory fitting accuracy. However, the ANN model had better predictive capacity than the UD model. Further, ANN-GA method had better performance than UD method for the iturin A yield optimization process. And more importantly, with higher optimization accuracy, the ANN-GA method needs much less experimental data than any other reported methods, which means the number of experiments can be significantly reduced. Thus, ANN-GA may be a better method, which can reduce the cost and increase the efficiency, for the large-scale optimization.

## Conclusions

In the present work, the focus was on using ANN-GA and UD to optimize iturin A production in flask-shaking fed-batch fermentation. For the first time, an ANN model based on UD data was established and comparisons between ANN-GA and UD were carried out. Three-factor-ten-level UD experimental data were used as the training set, and other 15 experimental data that were different from the training set were used as the test set. For the training set, the MRSE, correlation coefficient and error% of ANN model were 4.84, 100% and 0.039 respectively (compared with 32.21, 99.86% and 0.26 for UD). The satisfactory accuracy of the ANN fitting capacity indicated that the ANN model could work well based on the UD data, which had the minimal experimental number required to meet the needs of industrialization optimization. For the test set, the MRSE, correlation coefficient and error% of the ANN model were 237.58, 92.62% and 2.19 respectively (compared with 483.12, 78.58% and 4.15 of UD). Thus, ANN had better fitting, prediction and generalization capacities than UD based on the same experimental design. The sensitivity analysis indicated that when the perturbations in the input data occurred around the mean values, Asn addition was the most significant factor that had an effect on the iturin A yield, followed by Pro and Glu addition. Interestingly, when the perturbations were close to the edge of the range, the significance of Asn addition, followed by Glu and Pro, were comparable in the two models. Thus, although UD could get the insight information between variables directly, ANN was also demonstrated efficient in the sensitivity analysis. Additionally, ANN-GA was found to be more accurate in finding optimum conditions and predicting optimum iturin A titer. The optimum iturin A titer was 13364.5 ± 271.3 U/mL when the concentrations of added Asn, Glu and Pro were 155 mg/L, 320 mg/L and 160 mg/L, respectively. Further, using ANN-GA based on UD data, the iturin A titer was increased significantly by 34.6% (from 9929.0 ± 280.9 U/mL in batch fermentation without amino acids addition). Therefore, ANN methodology could be a better alternative to the UD approach based on the minimal experimental number that will reduce cost and increase efficiency in industrial applications.

## Material and methods

### Microorganism and medium

The *Bacillus subtilis* ZK8 strain that produces iturin A, was separated and mutated in our laboratory and stored in 4°C.

The slant culture medium contained 1.8 g/L MgSO_4_ · 7H_2_O, 1.5 g/L K_2_HPO_4_, 20 g/L peptone, 10 ml/L glycerol, and 1.8 g/L agar. The seed culture medium contained 25 g/L glucose, 30 g/L peptone, 2.86 g/L KH_2_PO_4_, and 3 g/L MgSO_4_. The fermentation culture medium was prepared with 31 g/L glucose, 3.8 g/L MgSO_4_, 0.79 g/L KH_2_PO_4_, 0.8 g/L yeast extract, and 2.4 g/L soybean protein powder hydrolysate.

### Fed-batch fermentation of iturin A

Strain ZK8 was activated in slant culture medium twice, and both cultures were incubated at 30°C for 36 h. The activated strain was then inoculated and incubated in the seed culture medium (100 mL culture in 500 mL flask) in a shaker at 30°C with 150 rpm for 20 h. Next, the seed culture was inoculated in 60 mL fermentation culture by 10% amount of inoculum for 48 h at 30°C with 150 rpm. After 24 hours of fermentation, three amino acids (Asn, Glu and Pro) were added to the broth in concentrations that were determined by statistical design. After fermentation, the broth was collected for further analysis.

### Iturin A yield evaluation: titer measurement

The yield of iturin A was determined by titer measurement which is the best way to evaluate the capacity of natural biosynthetic antibiotic like iturin A. Titer can elucidate the effects of antibiotics directly and indicate their likely field effects or clinical effects. The fermentation broth was centrifuged at 9000 rpm, and the supernatant was collected and sterilized as measured samples. The cylinder-plate method was used to measure the titer of iturin A. This is a typical method that is described in most pharmacopeia that has proved to be efficient in antibiotic evaluation [24-26]. *Phytophthora* sp. was used as the test organism.

### Experimental design

A three-variable ten-level uniform design was carried out to optimize the concentrations of three added amino acids in fed-batch fermentation. The uniform design table *U*_10_^*^(10^8^) was chosen for the experimental design. The concentration levels of independent variables *X*_*1*_ (Asn concentration, mg/L), *X*_*2*_ (Glu concentration, mg/L) and *X*_*3*_ (Pro concentration, mg/L) are shown in Table 7. The relationship between the responses and variables was expressed by a second order polynomial Eq. (1) as follows:

(1) $$Y=\beta_{0}+\sum_{i=1}^{m}\beta_{i}X_{i}+\sum_{i=1}^{m}{\beta_{i}}_{i}{X_{i}}^{2}+\sum_{i<j}\beta_{\mathrm{ij}}X_{i}X_{j}$$

where *Y* is the predicted response (iturin A titer, U/mL); *β*_*0*_ is a constant; *β*_i_, *β*_*ii*_ and *β*_*ij*_ are linear coefficient, square coefficient and cross coefficient respectively; and *X*_*i*_ and *X*_*j*_ are the independent variables.

**Table 7:** Factors and level values of uniform design

**Factor**	**Levels (mg/L)**									
	**1**	**2**	**3**	**4**	**5**	**6**	**7**	**8**	**9**	**10**
Asn(X_1_)	50	65	80	95	110	125	140	155	170	185
Glu(X_2_)	200	220	240	260	280	300	320	340	360	380
Pro(X_3_)	50	65	80	95	110	125	140	155	170	185

To estimate and avoid the system errors in the outputs, the experiments were repeated three times. The regression coefficients and ANOVA were calculated by Minitab software.

### Artificial neural networks

Common back-propagation (BP) artificial neural networks with a feed-forward structure were used to establish the predictive model for the iturin A titer. The ANN structure was composed of three layers with three neurons (one for each input variable) in the input layer and one neuron (iturin A titer) in the output layer. Several training algorithms (including “Fletcher-Reeves conjugate gradient”, “Ploak-Ribiere conjugate gradient”, “Powell-Beale conjugate gradient”, “BFGS quasi-Newton”, “one step secant”, “Resilient gradient descent”, “gradient descent”, “gradient descent with momentum”, “gradient descent with adaptive lr momentum” and “Levenberg-Marquardt Back Propagation” functions) were tested. For the hidden layer, a cut-and-try method was used to adjust the number of neurons from 3 to 12. The data were divided into two parts; the training set based on UD data, and the validation set, which included different patterns of the training set (shown in Tables 1 and 3). The aim of adjusting the structure was to obtain the fastest network convergence speed and the lowest mean-square-error (MSE), calculated using Eq. (2), between the predicted and actual outputs.

(2) MSE=1N∑i=1Nyi−yi'2

where *N* is the number of input values; *y*_*i*_ is the actual output value; and *y*_*i*_*’* is the predicted output value.

In this architecture, data always flows forward from input layer to hidden layer, and then to output layer. The adjustable parameter matrices, known as weights and biases, were associated with the connection between the nodes of the network. First the data were scaled by Eq. (3), and then the scaled input data were introduced to the hidden layer and summed using Eq. (4). The result was passed through the log-sigmoid activation function shown as Eq. (5), and became the input to the output layer. The activation function of the output layer was the linear function. The neuron in the output layer produced the output using the same procedure as the neurons in the hidden layer.

(3) $$X_{\text{scaled}}=\frac{{X-X}_{\text{min}}}{X_{\text{max}}-X_{\text{min}}}$$

where *X*_*scaled*_ is the scaled data matrix; *X* is the non-scaled data matrix; and *X*_min_ and *X*_max_ are the minimum and maximum values of the data matrices respectively.

(4) $$\text{net}=\sum_{i=1}^{N}X_{i}W_{i}+b$$

where *X*_*i*_ is the input data; *W*_*i*_ is the weight; and *b* is the bias.

(5) $$f\left(\mathrm{net}\right)=\frac{1}{1+\exp\left(‒\mathrm{net}\right)}$$

The ANN model was implemented in MATLAB 2012b, trained with the Levenberg-Marquardt back-propagation function, using the learning rate at 0.01. The training was stopped when the performance goal (MSE < 0.00001) was reached. Details of training an optimal ANN model with outstanding generalization capacity have been described in many reports [16,22,27].

### Sensitivity analysis

A sensitivity analysis was performed to find the effect of the input variables on the outputs. The effect of the addition of the individual amino acids can be captured in more obvious way in UD than in ANN. In the UD model, because of the regular form of variables in the quadratic equation, the importance and interactions of each variable can be read directly from the relative coefficients of the equation and ANOVA analysis. In the ANN model, which is performed as a ‘black box’, the information is not given directly, so other methods need to be used to assess its sensitivity analysis. In this study, a perturbation analysis [28,29] based on the mean value was chosen for the sensitivity analysis [30]. The sensitivity of each variable was evaluated by the standard deviation of the net output perturbation caused by the perturbation of one input variable when the other variables were constant, according to Eq. (6) as follows:

(6) $$S_{i}=\sqrt{\frac{\sum_{j=1}^{n}f{\left(X_{i}+\epsilon_{j},W\right)}^{2}}{n}}\;-\sigma_{i}\leq \epsilon_{j}\leq \sigma_{i}\;j=1,2,\ldots ,n$$

where *S*_*i*_ is the sensitivity estimating value of input on the output function; *X*_*i*_ is the variables; *ϵ*_*j*_ is the random value among the deviation area; *σ*_*i*_ is the standard deviation of input data; n is the number of random values, which was 30 in this case; *W* is the weight in the ANN model; and *f*(*X*,*W*) is the output of the ANN model.

### Optimization by GA

GA was carried out to optimize the input area of the ANN model with the aim of maximizing the iturin A titer. The GA program was implemented in MATLAB, with the real-coded chromosome length = 1, population size = 20, crossover probability = 0.4, mutation probability = 0.2, and max generations = 100. The ANN model was used as the fitness function of GA. And the GA optimizing procedure was run several times with different random initialized conditions.

For UD, optimization was achieved by taking the partial derivative of the function (*Y*) with respect to the variable (*X*).

## Abbreviations

(ANN): Artificial neural network; (UD): Uniform design; (RSM): Response surface methodology; (GA): Genetic algorithm; (Asn): Asparagine; (Glu): Glutamic acid; (Pro): Proline; (MSE): Mean-square-error; (RMSE): Root-mean-square-error; (BP): Back propagation.

## Competing interests

The authors declare that they have no competing interests.

## Authors' contributions

WP carried out the fed-batch fermentation. WP, JZ, YR, JY, TX and JZ established the analytical method of yield measurement. WP participated in the ANN and GA program writing. WP, YR, TX and SX participated in the UD experimental design. WP, JY and JZ participated in the statistical analysis. WP and JZ carried out the validation and verification experiments. WP, YR and TX were responsible for the methods comparison. HT conceived of the study, and participated in its design and coordination and helped to draft the manuscript. All authors read and approved the final manuscript.
